# Pharmaceutical profile and prescribing practices in compounding pharmacies in Brazil: a cross-sectional study

**DOI:** 10.3389/fphar.2026.1893112

**Published:** 2026-08-28

**Authors:** Gustavo Pereira Calado, Débora Santos Lula Barros, Rodrigo Fonseca Lima, Rafael Santos Santana

**Affiliations:** Laboratory of Evidence and Pharmaceutical Studies (LEFAR), Department of Pharmacy, Faculty of Health (FS), University of Brasília (UnB), Brasília, Brazil

**Keywords:** drug compounding, drug utilization review, health knowledge, pharmaceutical services, prescriptions

## Abstract

**Introduction:**

The industrialization expanded drug access but standardized formulations often fail to meet individual clinical specificities. Compounding pharmacies fill this gap by providing personalized therapies. In Brazil, although Federal Pharmacy Council Resolutions grant pharmacists the autonomy to prescribe over-the-counter drugs and compounded formulations, this clinical practice faces legal instability and structural challenges. This study aimed to outline the profile of pharmacists and analyze the clinical practice of pharmaceutical prescribing in compounding pharmacies across Brazil.

**Methods:**

This is a quantitative, descriptive, and cross-sectional study conducted through an online survey covering all 27 federal units in Brazil. The questionnaire was distributed between May and December of 2024 via institutional channels and professional social networks. The sample consisted of 186 licensed pharmacists working in the compounding sector. Data collection addressed personal and professional information, prescription practices, and the management of self-limiting health problems.

**Results:**

The sample was predominantly female (78.5%), with a high concentration in the Southeast region (35%). While 73.1% of participants perform pharmaceutical prescribing, only 43.4% document these prescriptions according to regulatory requirements. Furthermore, 44.3% of participants reported never having taken training courses related to prescribing. The most frequently managed health demands included mild insomnia (86.6%), weight loss (84.4%), and anxiety (83.9%). Herbal medicines were the most prescribed class (87.7%), with *Passiflora incarnata* (64.8%) being the most common active ingredient. Major barriers to prescribing included a lack of time (67.6%), absence of financial incentives (36.3%), and inadequate infrastructure (32.4%). Most professionals (61.8%) provide prescribing services free of charge.

**Discussion:**

Pharmacists in Brazilian compounding pharmacies actively perform clinical prescribing, primarily managing self-limiting conditions with herbal and nutritional therapies. However, a significant gap persists between practice and formalization, characterized by low documentation rates, lack of infrastructure privacy, and training deficiencies. Addressing these regulatory, educational, and structural limitations is critical to ensuring patient safety, establishing clinical governance, and consolidating the role of the compounding pharmacist in the healthcare system.

## 1 Introduction

The mass production of medicines in the Industrial Revolution changed the dynamics of the pharmaceutical industry by expanding and democratizing the population’s access to drugs. However, despite the expansion of the market, compounding pharmacies remained a viable alternative to meet therapeutic demands that are not covered by the industry’s standardized formulations. Patients with specific needs, such as those who are intolerant, allergic, or belong to vulnerable groups, tend to resort to compounding services as a way of obtaining individualized care (Tischner et al., 2024).

This scenario highlights a limitation of commercial formulations, given that the standardization of pharmaceutical forms does not always correspond to the clinical specificities of each patient. It is often necessary to adjust dosages and concentrations, as well as pharmaceutical forms and presentations, according to the particularities of each patient. Through compounding pharmacies, it is feasible to prescribe and strictly follow the prescriptions tailored to one’s specific needs (Dias et al., 2020).

Considering this relevance, Brazilian Law number 13.021/2014 defined pharmacy as a health establishment intended to provide essential pharmaceutical services, including the compounding and dispensing of magistral, officinal, pharmacopeial, or industrialized medicines, in addition to pharmaceutical supplies and products (Presidency of the Republic, 2014).

In this context, compounding pharmacies reaffirm their importance by offering personalized and individualized medicines based on clinical, pharmaceutical, or medical requests on a small scale, unlike drugstores, which sell industrialized medicines (Júnior and Rodrigues, 2023). Thus, these pharmacies represent not only the continuity of the traditional practice of compounding, but also the clinical role of the pharmacist, as well as the innovation and the social responsibility shown within the sector (Tischner et al., 2024).

Among the clinical duties of pharmacists, the prescription of medicines stands out as a fundamental tool in promoting the rational use of medicines and preventing self-medication (Santos et al., 2022). Prescribing requires technical, scientific, and clinical knowledge, as well as communication skills to guide patients on the safe and effective use of the proposed drug treatment. Despite this, pharmacists’ adherence to prescribing practices is still scarce, which may be related to different factors, such as the pharmacist’s own perception of their clinical role, their level of training, the perceived requirements for prescribing, and working conditions (Ramos et al., 2022).

The practice of pharmaceutical prescription of compounding medicines is supported, at a global scale, by regulatory frameworks that balance clinical autonomy with strict safety and quality standards. In Canada, regulation is decentralized to provincial bodies (Colleges of Pharmacy) that adopt the technical standards of the National Association of Pharmacy Regulatory Authorities (NAPRA), which allow prescription models that range from the management of minor conditions to Additional Prescription Authorizations (APA) (National Association of Pharmacy Regulatory Authorities, 2015; Health Canada, 2009). In England, the right to compound is guaranteed by the Human Medicines Regulations, and clinical practice is governed by the General Pharmaceutical Council (GPhC), which licenses professionals as independent prescribers, allowing them to prescribe any medicine within their clinical competence (Nobbs, 2020; Hussain and Babar, 2023).

In Brazil, the norms that govern clinical practice, based on Federal Pharmacy Council Resolutions number 585 and number 586, of 2013, regulate the clinical duties of pharmacists, granting them autonomy to prescribe over-the-counter drugs (OTCs) as well as medicines previously approved by ANVISA (Federal Council of Pharmacy, 2013a; Federal Council of Pharmacy, 2013b; Freitas et al., 2022). The autonomy of pharmaceutical prescribing in Brazil is undergoing a period of legal instability, which is marked by a dispute over professional competencies with the medical profession. Recent court decisions have suspended the effects of Federal Pharmacy Council Resolutions number 586/2013 and number 5/2025, on the understanding that such norms exceed regulatory power by conflicting with exclusive acts established by the Medical Act Law (Law number 12.842/2013). Currently, the validity of these prerogatives is *sub judice*, which creates uncertainty for practicing professionals and reinforces that the definitive consolidation of this clinical attribution needs to be validated by the National Congress (Federal Council of Pharmacy, 2025; Presidency of the Republic, 2013).

Given the growing demand for personalized therapies, pharmaceutical prescribing has become a strategic clinical tool in compounding pharmacies. However, a critical knowledge gap remains regarding how prescribing is integrated into daily practice within this sector. While previous studies focused on general community pharmacies or regional samples, this is the first nationwide survey across all 27 Brazilian federal units dedicated to compounding pharmacies. Therefore, this study aims to fill this gap by characterizing clinical prescribing practices and identifying key predictors and barriers influencing pharmaceutical prescribing in compounding pharmacies in Brazil.

## 2 Methods

This is a quantitative, descriptive and cross-sectional study that was conducted through an online survey covering all federal units of Brazil. The data collection instrument was developed based on clinical guidelines from Evidence-Based Pharmacy (Santana and Reis, 2024). Content validation was conducted by an external panel of five expert pharmacists independent of the study team, specializing in clinical pharmacy, compounding practices, and pharmacoepidemiology. Evaluators independently reviewed each questionnaire item for clarity, relevance, and clinical applicability using a structured evaluation form, and consensus was achieved through a two-round iterative feedback process where items were refined until full agreement was reached. Following expert consensus, a pilot study was conducted with a sample of 10 licensed compounding pharmacists to pre-test the questionnaire. This stage assessed overall comprehension, logical flow, and completion time (averaging 10–12 min). Minor wording adjustments were made based on pilot feedback, and pilot responses were excluded from the final study analysis.

The questionnaire was sent to pharmacists between May and December of 2024, using a multifaceted dissemination strategy to reach pharmacists in all regions of Brazil. Dissemination was carried out through institutional channels, including getting in contact with professional associations (Regional Pharmacy Councils and the Federal Pharmacy Council).

Additionally, a strategy of active recruitment and direct contact with participants was employed through professional Instagram, WhatsApp, and LinkedIn accounts. On LinkedIn, an active search was conducted using search filters by position (Pharmacist; Pharmaceutical; Magistral; Compounding) and segmentation by state, allowing for individualized invitations to professionals from different federal units. On WhatsApp, prospecting took place in technical groups and pharmacists’ communities. On all platforms, the author sent direct messages explaining the research objectives and eligibility criteria and requesting the completion of the electronic form, ensuring access to the Free and Informed Consent Form (known as TCLE, in Portuguese).

To ensure the integrity and reliability of the data collected, a participant screening process was adopted. Professionals who met the following inclusion criteria were considered eligible: (I) complete degree in pharmacy; (II) active registration with the Regional Pharmacy Council; and (III) professional practice in compounding pharmacies (public or private). Eligibility was validated through a data verification stage, in which the professional registration information provided by the respondents was cross-checked with the official databases of the Regional Pharmacy Councils, by using the registration query systems. This procedure allowed us to confirm the active status of the registration and the accuracy of the professional identification, which ensured that the sample was composed strictly of licensed pharmacists in full exercise of their duties. Additionally, to prevent duplicate submissions, survey settings required unique Google account authentication, and professional registration numbers (CRF) were systematically cross-referenced during data cleaning to identify and eliminate any potential duplicate entries.

The sample size was determined based on an estimated finite population of approximately 20,000 pharmacists working in the Brazilian compounding sector, according to indicators of active establishments from the Federal Pharmacy Council for the year of 2024, and taking into account the existence of approximately 10,000 compounding pharmacies in Brazil and at least two pharmacists working in each of them, according to data from the sector overview of the National Association of Compounding Pharmacies (ANFARMAG) (Federal Council of Pharmacy, 2026; National Association of Compounding Pharmacists, 2026; Ferres et al., 2025). Adopting a confidence level of 95% (Z = 1.96) and a proportion of 50%, the final sample of 186 participants gave the study, although non-probabilistic convenience sampling limits strict national representativeness, geographic coverage across all 27 Brazilian federal units enhances the national scope and diversity of the analyzed cohort. Furthermore, calculated statistical estimates and bivariate associations reflect unadjusted comparisons inherent to the non-probabilistic design.

The instrument was developed based on the clinical guidelines of the Evidence-Based Pharmacy book (Santana and Reis, 2024), from the Laboratory of Evidence and Pharmaceutical Studies at the University of Brasília (Lefar-UnB). The questionnaire was made operational through Google Forms, chosen for its accessibility and practicality, and organized into three thematic blocks: (I) personal data; (II) professional information; and (III) data on prescription and management of self-limiting health problems. For the purpose of this study, pharmaceutical prescribing was operationally defined in accordance with CFF Resolutions No. 585/2013 and 586/2013 as the clinical practice through which pharmacists select and recommend OTC drugs, herbal products, dietary supplements, or compounded formulations to manage self-limiting conditions. Rather than providing prior restrictive instructions to respondents, the survey captured the distinction between formal documented prescribing and informal verbal advice through its design: participants first reported whether they performed prescribing activities, and practicing prescribers were subsequently asked whether these clinical recommendations were formally documented in writing according to regulatory standards.

Among the aspects investigated, professional information was evaluated, including workload, monthly pay, and the pharmaceutical services performed. With regard to pharmaceutical prescribing, not only the practice itself was analyzed, but also other factors, such as the level of knowledge of the professionals involved, the existence of a pharmaceutical office in the establishment, the receipt of commissions for prescriptions or pharmaceutical services, the average value of the commission received, and the average amount charged to the patient for the prescription. Regarding the management of health problems, we were able to identify the main complaints that motivate care, the most prescribed classes of medications, the therapeutic purposes of the most prescribed herbal medicines, as well as the daily frequency of herbal medicine prescriptions and other magistral active ingredients, adopting a periodicity scale ranging from “never prescribed” to “very often prescribed.” Additionally, participants were asked about the perceived barriers to implementing prescriptions and the impacts of this practice on the pharmacy. Finally, the participants’ perceptions regarding the need for specific clinical guidelines for patient care in compounding pharmacies were investigated.

This research was conducted in strict compliance with the ethical precepts governing research involving human subjects in Brazil. The protocol was approved by the Research Ethics Committee of the Faculty of Health Sciences of the University of Brasília (CEP/FS-UnB), under favorable opinion number 5.171.224, in accordance with National Health Council Resolution number 466/2012. Participation was voluntary and conditional upon reading and formally accepting the Free and Informed Consent Form, available in digital format on the form’s home page. Participants were advised to keep a copy of the form, which could also be sent by email upon individual request.

Descriptive statistics were used to summarize categorical variables as absolute frequencies and percentages. To provide deeper analytical insight into pharmaceutical prescribing practices, bivariate inferential statistics were conducted. Associations between categorical predictors (e.g., specific training courses, professional experience, presence of a private consultation room) and primary outcomes (e.g., practicing prescription, formal documentation, charging/receiving commissions, and self-reported high knowledge) were evaluated using Pearson’s Chi-square test ( $\chi^{2}$ ) or Fisher’s exact test when expected cell counts were less than 5. Odds Ratios (OR) with 95% Confidence Intervals (95% CI) were calculated to estimate the strength of associations. A two-tailed p -value <0.05 was considered statistically significant. All statistical analyses were performed using Jamovi (version 2.4, The jamovi project). Bivariate analyses were performed using Chi-square tests and unadjusted Odds Ratios (OR) with 95% Confidence Intervals (95% CI) to examine unadjusted associations between demographic/professional variables and primary outcomes (prescribing practice and formal documentation). A comprehensive summary of all tested variables, reference categories, denominators, unadjusted ORs, and non-significant results is provided in Supplementary Table S1.

A total of 186 licensed pharmacists participated in the study. The effective sample size ( n ) varies slightly across specific variables due to data cleaning procedures, in which incomplete or inconsistent responses were excluded, as well as item-nonresponse arising from optional survey fields. All corresponding denominators and valid response totals are explicitly specified in the text, tables, and figures.

## 3 Results

The sample consisted of pharmacists from the 27 federal units in Brazil, with a predominance of females (78.5%). Geographically, the highest concentration of participants was in the Southeast region (35%), followed by the Northeast region (27%), with the state of São Paulo standing out with 18.9% of the total representation. Regarding occupational profile, 53.23% (n = 99) of professionals reported working 44 h per week, while 20.9% (n = 39) reported having worked in the field for between 6 and 10 years. In terms of pay, 45.7% (n = 85) of participants reported earning between R$3,000 and R$5,000 per month, while 29.6% (n = 55) earned between R$5,000 and R$7,000.

In relation to the pharmaceutical services performed, the dispensing of compounded medicines stood out (72%), followed by the pharmaceutical prescription of compounded formulas and the management of compounding laboratories (67.6%). However, when analyzing complementary training for activities such as pharmaceutical prescribing, it was found that 44.3% of participants had never taken training courses.

In regards to the practice of pharmaceutical prescribing, it was found that 73.1% (n = 136) of participants perform this clinical task, but only 43.4% (n = 59) of these document prescriptions in accordance with the requirements of Resolution number 586/2013 of the Federal Pharmacy Council (CFF, in Portuguese). Meanwhile, 25.8% (n = 48) do not issue pharmaceutical prescriptions in compounding pharmacies. In addition, 47.3% (n = 88) of participants reported having average knowledge about prescribing medicines, while another 36.5% (n = 68) stated that they have a high level of knowledge and apply it daily. In contrast, 13.4% (n = 25) stated that they had little knowledge and did not use it in their day to day.

Bivariate inferential analysis revealed key predictors of pharmaceutical prescribing practices and formal documentation in Brazilian compounding pharmacies. Professional training in pharmaceutical prescribing was strongly associated with practicing prescription (χ2 = 22.71, p < 0.001), with trained pharmacists being nearly six times more likely to prescribe than non-trained peers (OR = 5.87, 95% CI: 2.75–12.53, p < 0.001). Furthermore, experience over 5 years significantly increased the likelihood of prescribing (OR = 2.43, 95% CI: 1.16–5.09, p = 0.019).

Among pharmacists who prescribe (n = 136), the presence of a dedicated, private pharmaceutical consultation room was a major predictor of formal prescription documentation (χ2 = 8.86, p = 0.003). Pharmacists operating in private consultation rooms had a 3.29-fold higher chance of documenting their prescriptions (OR = 3.29, 95% CI: 1.51–7.16, p = 0.003) compared to those working at the pharmacy counter. Additionally, the availability of a consultation room significantly increased the likelihood of receiving commissions or charging for clinical services (OR = 4.50, 95% CI: 1.87–10.81, p < 0.001).

When asked about the existence of a pharmaceutical consultation room, it was found that a minority of 28.5% (n = 53) had an adequate space, as established by Resolution number 585/2013 of the CFF, which requires an area with physical conditions that guarantee the confidentiality and dignity of the patient. From another perspective, the data reveal that 61.3% (n = 114) provide care and guidance directly at the pharmacy counter, which compromises patient access and openness.

**FIGURE 1:**
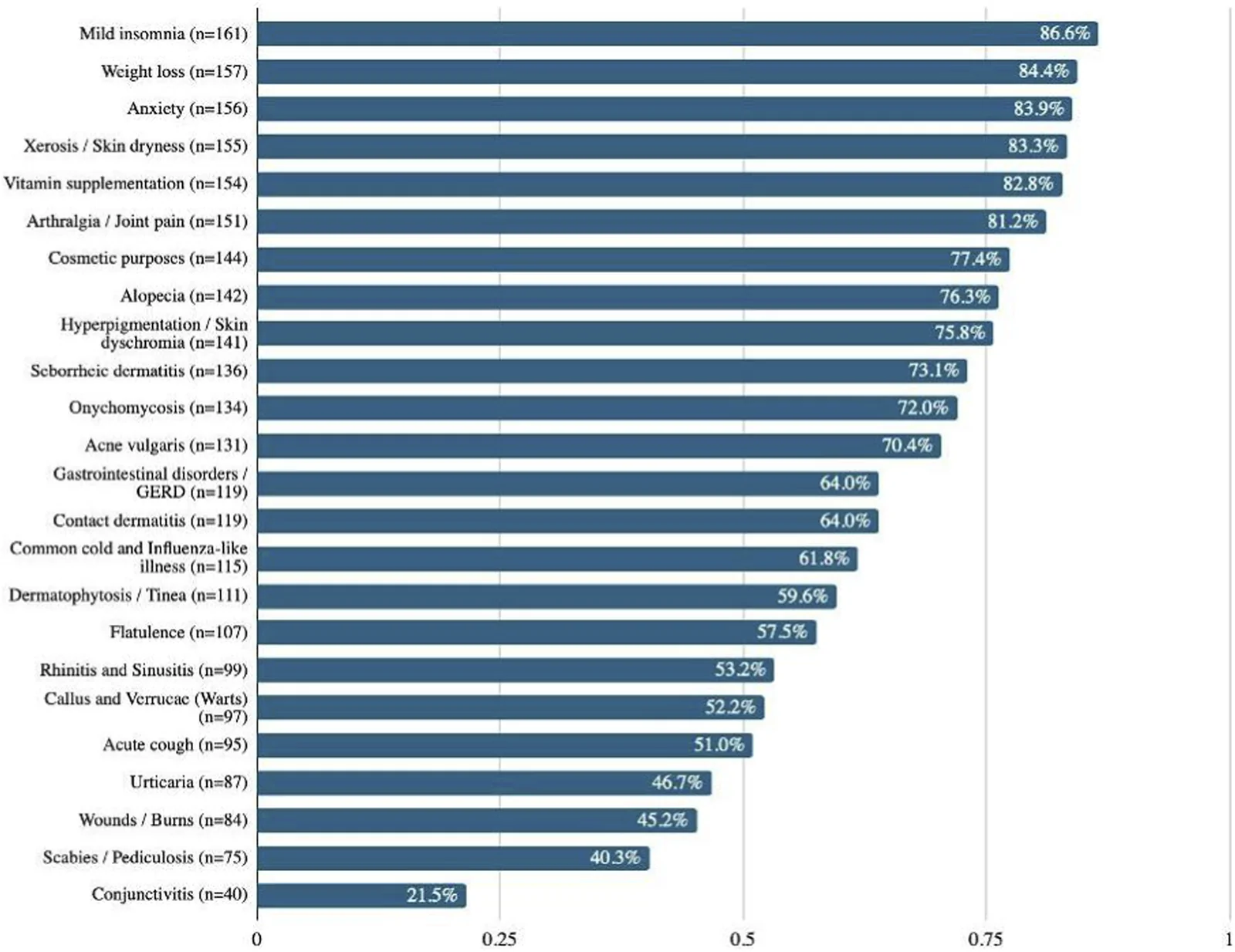
Health problems most frequently managed by pharmacists in compounding pharmacies. Federal Council of Pharmacy (2025), N = 186. Source: Survey data (2025).

The health problems that lead patients to seek pharmaceutical care in compounding pharmacies were also evaluated, with a predominance of demands related to mild insomnia, weight loss, anxiety, dry skin, and vitamin supplementation with significant daily and weekly frequencies. In contrast, diseases such as conjunctivitis, scabies, pediculosis, skin burns, urticaria, and acute cough indicated the lowest rates of management (Figure 1).

**FIGURE 2:**
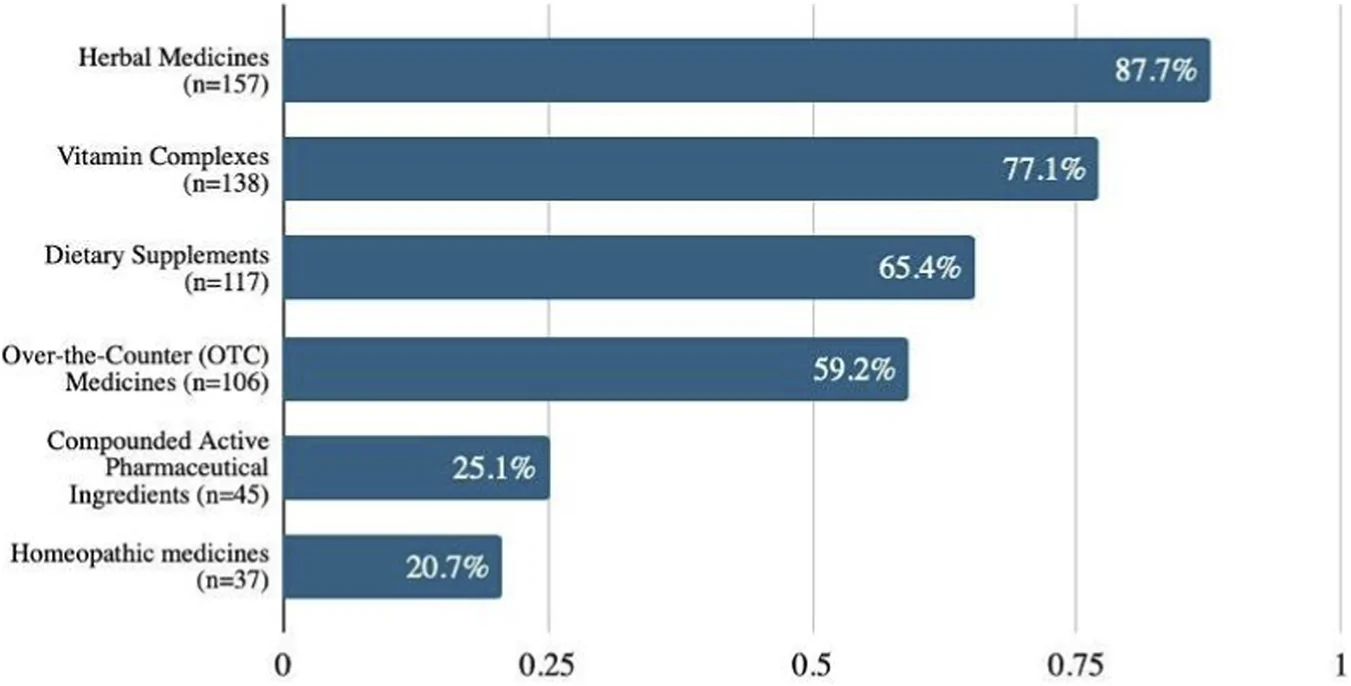
Classes of drugs most frequently prescribed by pharmacists in compounding pharmacies. Federal Council of Pharmacy (2025), n = 179. Note: Sample size (n = 179) reflects valid responses for this question out of the total study sample (N = 186). Source: Survey data (2025).

The classes of medications most frequently prescribed by pharmacists in compounding pharmacies are listed in Figure 2. Herbal medicines had the highest prevalence among the classes (87.7%), followed by vitamin complexes (77.1%) and dietary supplements (65.4%). The prescription of OTCs, pharmaceutical products that do not require a prescription because they are intended for the treatment of mild symptoms and disorders and have proven safety and efficacy (National Health Surveillance Agency, 2016), occurred in 59.2% of cases. Homeopathic medicines had the lowest prescription frequency (20.7%).

**FIGURE 3:**
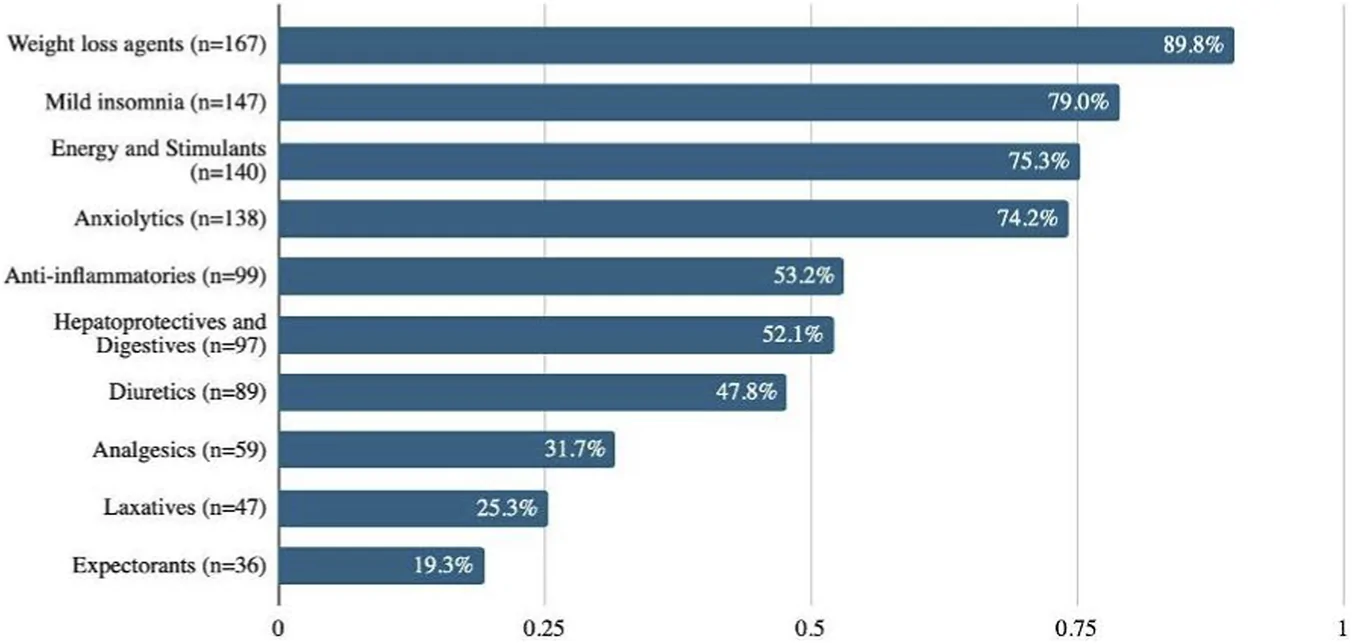
Most requested clinical indications for herbal medicine prescriptions in compounding pharmacies. Federal Council of Pharmacy, 2025, n = 186. Source: Survey data (2025).

Considering the high frequency of herbal medicine prescriptions, the main clinical indications for these interventions were investigated, with emphasis on weight loss (89.8%), management of mild insomnia (79.0%), and use for stimulant and energy-boosting effects (75.3%) (Figure 3).

**FIGURE 4:**
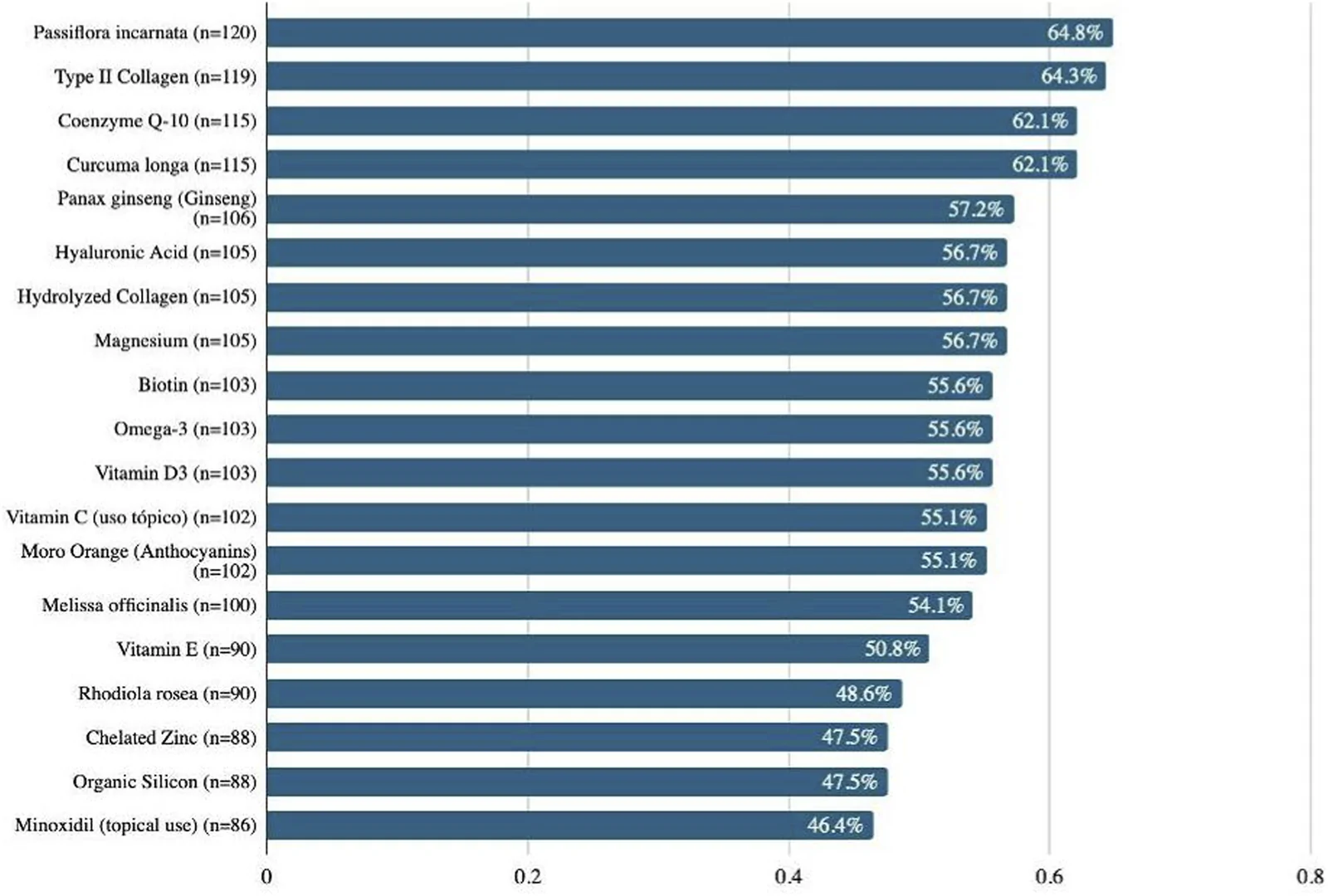
Magistral active ingredients (isolated substances and herbal medicines) and herbal medicines most frequently prescribed by pharmacists in compounding pharmacies. Federal Council of Pharmacy, 2025, n = 185. Note: Sample size (n = 185) reflects valid responses for this question out of the total study sample (n = 186). Source: Survey data (2025).

The study evaluated the frequency of prescriptions for active ingredients and herbal medicines in the compounding setting (Figure 4). In the most recurrent stratum (“highly prescribed”), *Passiflora incarnata* (64.8%) and Type II Collagen (64.3%) stood out, followed by *Curcuma longa* and Coenzyme Q-10 (both with 62.1%).

When asked about the daily frequency of prescriptions (n = 179), it was found that most professionals (52.5%; n = 94) reported that they write one to five prescriptions daily, while 19% (n = 34) write between six and twenty prescriptions per day. The limitations faced by pharmacists in prescribing reveal a multifactorial scenario that restricts their full clinical practice. Although on a smaller scale, aspects such as the perception of low demand, technical and professional insecurity, and demotivation caused by reduced patient adherence were identified. Professionals also report that pharmaceutical prescribing can have positive repercussions in different dimensions, both for compounding pharmacies, improving profitability and financial performance, and in improving the wellbeing and quality of life of patients, solving self-limiting health problems and promoting the rational use of medicines (Table 1).

**TABLE 1:** Potential barriers to pharmaceutical prescribing in the management of self-limiting health problems (n = 179) and professionals’ perceptions of its positive impacts on compounding pharmacies (n = 182). Federal Council of Pharmacy, 2025.

Barriers (n = 179)	*n*	%	95% CI
Lack of time due to other technical and administrative activities	121	67.6	60.7–74.5
Lack of financial incentives	65	36.3	29.3–43.3
Lack of a suitable location to perform the service	58	32.4	25.5–39.3
Lack of support from the company	40	22.3	16.2–28.4
Limited human resources	37	20.7	14.8–26.6
Does not feel qualified to write a prescription	23	12.8	7.9–17.7
Does not feel confident prescribing medications	19	10.6	6.1–15.1
Benefits (n = 182)			
Improvement in patients’ quality of life	174	95.6	92.6–98.6
Enhancement of the pharmaceutical profession	158	86.8	81.9–91.7
Increase in company profits and profitability	147	80.8	75.1–86.5
Promotion of rational use of medicines	147	80.8	75.1–86.5
Improvement of symptoms and resolution of self-limiting health problems	115	63.2	56.2–70.2
Expansion of prescribing options for pharmacists	115	63.2	56.2–70.2
Avoid overload in hospital settings	86	47.3	40.4–54.6
Reducing costs for the Unified Health System	76	41.8	34.6–49.0

Sample sizes (n = 179 for barriers; n = 182 for benefits) reflect valid responses for each item out of the total study sample (n = 186). Source: Survey data (2025).

The study also shows that most participants (61.8%; n = 115) provide pharmaceutical prescriptions free of charge, that is, without charging for the service provided. A smaller portion (10.7%; n = 20) receives some type of pay based on a percentage of the compounded medicines and only (3.7%; n = 7) charge per consultation or service provided. In addition, 23.1% (n = 43) of participants do not perform this type of service.

Most professionals who perform pharmaceutical prescriptions (54.8%; n = 102) do not receive any type of commission for the service. Only a minority reported receiving pay, with 4.3% receiving between 1% and 5%, and another 4.3% receiving between 5% and 10%. Higher percentages were identified, with 1.1% receiving between 10% and 15%, while 1.6% receive between 15% and 20%. In contrast, 1.1% receive less than 1% commission based on the value of the compound prescription. Regarding the average nominal value charged per prescription, 3.7% (n = 7) of participants reported charging between R$5.00 and R$40.00 per prescription.

The analysis of pharmacists’ perceptions of the need for specific clinical guidelines reveals that most participants (75.8%; n = 141) considered the development of clinical protocols and guidelines to be very necessary. The option “partially necessary” was selected by 18.8% (n = 35) of the sample, while 2.6% (n = 5) considered it not to be very necessary.

## 4 Discussion

The analysis of the study’s sociodemographic profile shows a predominance of female professionals with high workloads and average compensations. The results also reveal a predominantly female workforce (78.5%), which reflects a national trend toward the feminization of health professions, as corroborated by data from the Federal Pharmacy Council, which indicate that women represent about 70% of registered professionals in the country (Ramos et al., 2022; De Souza et al., 2022; Santi, 2015). These data have implications for labor dynamics because, despite being the numerical majority, female pharmacists still face significant barriers in advancing to senior leadership positions and wage disparities (Santi, 2015).

The study reports that, in addition to dispensing compounded medicines, the other prevalent clinical activity is the prescription of magistral medications. However, there is a gap between the practice of prescribing and its formalization, given that while 73.1% of pharmacists claim to prescribe, only 43.4% document their prescriptions as required by CFF Resolution number 586/2013. This informal prescribing represents a vulnerability for patient safety and for the consolidation of clinical pharmacy in Brazil. In parallel with other studies, other typical clinical services of community pharmacies were presented, such as pharmacotherapy review, therapeutic monitoring, health education, and self-care support in minor conditions (López and Gallegos, 2022; Nassur et al., 2020; Garabeli et al., 2022; Rotta et al., 2023).

The high adherence to prescription practices in compounding pharmacies (over 70%) contrasts with the low rate of formal documentation, which represents a challenge to the advancement of pharmaceutical practice as a systematized process. CFF Resolution number 586/2013, which regulates pharmaceutical prescriptions in Brazil, highlights the need for adequate documentation and good clinical practices, but its implementation varies in practice. Documentation is not a mere bureaucratic requirement; it is fundamental to the continuity of patient care and safety. Studies show that in a healthcare system where patients move between different levels of care, an undocumented prescription is lost information, which can lead to therapeutic duplications or drug interactions undetected by other prescribers (Kim et al., 2024).

Furthermore, with regard to professional training, a significant proportion of pharmacists (44.3%) have not taken any training courses on the subject, which shows that the lack of specific training, both initial and continuing, still represents a limiting factor for the performance of these professionals. Reviews on pharmaceutical prescribing highlight that competence and confidence in prescribing are critical issues even in international contexts, with studies showing that professionals may feel a lack of formal preparation despite their technical skills in pharmacotherapy (Woit et al., 2020). This discrepancy between knowledge and confidence highlights the need to strengthen clinical education at undergraduate and graduate levels, aligning it with the real demands of pharmaceutical practice.

The observed care infrastructure, with a predominance of over-the-counter care and low availability of pharmaceutical consultation rooms, reflects a challenge for the consolidation of clinical practice in compounding pharmacies. Pharmaceutical care emphasizes that adequate environments and privacy are essential elements for quality pharmaceutical practice, by favoring being welcoming to the patient, active listening, and appropriate clinical decision (Mastroianni et al., 2023). This scenario suggests that the transition from a commercial model to a patient-centered model is still underway.

Privacy remains a fundamental determinant for effective pharmaceutical care. Evidence shows that 56.2% of patients avoid clinical discussions with the pharmacist due to inadequate confidentiality (Alrasheed et al., 2024). Qualitative studies show that patients in community pharmacies often withhold information about adherence, side effects, or stigmatizing conditions when care is provided in public or semi-private areas, for fear of being overheard by others. Inadequate physical and acoustic privacy ultimately devalues consultations into commercial transactions and compromises intervention effectiveness (Dhital et al., 2022; Hattingh et al., 2016). The lack of dedicated consultation rooms perpetuates the pharmacy’s commercial image. To consolidate pharmaceutical prescribing, infrastructure must align with evolving clinical roles, as privacy remains a critical barrier to patient engagements (Alghamdi et al., 2023).

The findings regarding the most frequently managed clinical demands, such as insomnia, anxiety, weight loss, and dermatological and aesthetic problems, are consistent with studies that identify that pharmacists act more comprehensively in self-limiting conditions or those related to the promotion of wellbeing, especially in community services (Brandão and Santos, 2025; Dagnew et al., 2026; Selam et al., 2022). This demonstrates that compounding pharmacies play an important role in the management of common complaints, although there is a need to broaden the scope to deal with more complex clinical conditions, always supported by evidence and well-established protocols.

Our findings regarding the frequent prescription of herbal and OTC products closely align with broader national data on pharmacist prescribing in Brazil. Milani et al. (2024) observed that ambulatory prescribing remains largely concentrated in OTC medications (42.0%), with minimal integration into collaborative protocols. Similarly, our results reflect a daily clinical practice primarily driven by self-care demands, such as weight management and mild symptom relief. However, Milani et al. (2024) also exposed persistent systemic hurdles, including infrastructure gaps, training disparities, and interprofessional friction with physicians. Contextualizing our data within this national scenario reveals a dual reality: while Brazilian pharmacists actively embrace prescriptive autonomy, especially in phytotherapy and compounding, fully realizing this potential requires structured collaborative frameworks, standardized clinical protocols, and targeted training to ensure safe, evidence-based care.

The high frequency of prescriptions for herbal medicines and compounded drugs in the study corroborates the trend observed in the Brazilian literature of combining traditional and complementary therapies as part of comprehensive patient care (Izidoro, 2025). However, the scientific evidence for many herbal medicines varies, and the international literature points to a growing role for pharmacists in integrating these therapies when supported by robust data on efficacy and safety. Studies of herbal medicines in compounding pharmacies also show a predominance of herbal anxiolytics and cholagogues/digestives (Nardon et al., 2024).

The high prevalence of Moro orange (*Citrus sinensis*) prescriptions for weight loss highlights a gap between compounding practice and robust clinical evidence. Although anthocyanin-rich Moro orange extracts demonstrate statistically significant reductions in body weight and fat mass (Briskey et al., 2022; Campos et al., 2025), current evidence remains limited by a small number of randomized controlled trials (RCTs) with small sample sizes, such as a meta-analysis pooling only three RCTs (n = 252) (Campos et al., 2025). Moreover, modest weight reductions (e.g., ∼2 kg) raise questions regarding long-term clinical relevance and sustainability without lifestyle changes. Key parameters such as optimal dosing, bioequivalence, and long-term safety also remain uncharacterized. Thus, while promising as an adjuvant therapy, Moro orange requires critical evaluation by prescribers and larger, independent RCTs to support evidence-based practice.

Studies conducted in countries such as the United Kingdom, Canada, and Australia demonstrate that the consolidation of pharmaceutical prescribing was directly associated with the adoption of structured models of clinical governance, rigorous documentation requirements, and the incorporation of evidence-based guidelines, elements that favored both patient safety and institutional recognition of the pharmacist as a prescriber (Cope et al., 2016; Ghabour et al., 2023).

Barriers to pharmaceutical prescribing, such as lack of time, and absence of financial incentives and institutional support, are consistent with evidence identifying organizational factors as determinants for the effective implementation of prescribing by pharmacists (Lugo et al., 2019). Furthermore, they indicate that, although pharmaceutical prescribing improves access to medicines, structural barriers persist and need to be addressed, such as the fragility of specific regulations for pharmaceutical prescribing and the expansion of clinical scope, which generates legal uncertainty and limited action (Elshazly et al., 2025) and changes in compensation models, training, and integration with health systems (Walpola et al., 2024).

Crucially, these structural, regulatory, and training barriers are not exclusive to Brazil; they closely mirror the challenges faced across Europe. Research in the European context shows that pharmaceutical compounding often suffers from a lack of harmonized laws among different countries, which fragments professional standards (Minghetti et al., 2014). Furthermore, a recent review on expanded pharmaceutical services across European and OECD countries confirmed that time constraints, workload pressures, and lack of financial incentives remain major hurdles to implementing clinical care in pharmacies (Karia et al., 2024). This international comparison highlights that moving toward a clinically centered compounding practice is a global challenge that requires stronger institutional support and standardized guidelines to ensure patient safety worldwide.

The impacts perceived by participating pharmacists, such as professional recognition and improved quality of life for patients, corroborate studies showing that the integration of pharmacists into prescribing can improve health indicators and strengthen primary care, especially in healthcare settings. In countries with well-established models of pharmaceutical prescribing, there are measurable benefits in terms of treatment adherence and therapeutic optimization (Mesbahi et al., 2025).

The issue of compensation reveals a tension between clinical responsibility and insufficient financial recognition, which is described as a constraint on the sustainability of clinical pharmacy practice in many contexts. Without clear mechanisms for payment for clinical services, prescribing practice may continue to depend on individual initiatives rather than becoming an integral part of health policies (Ramos et al., 2022). Furthermore, commercial incentives, such as receiving commissions, highlight a critical practice-level conflict of interest, as compounding pharmacists may simultaneously evaluate, prescribe, prepare, and sell the medication. This overlapping role risks introducing financial bias that could compromise clinical objectivity and rational drug use.

Pharmacists’ strong perception of the need for specific clinical guidelines for compounding pharmacies reflects the regulatory and technical gap that still exists in this practice in Brazil. Other studies have already reported that the development and adaptation of contextualized clinical protocols are fundamental for patient safety and for the institutional recognition of pharmaceutical prescribing as an evidence-based practice (Ramos et al., 2022). In several developing countries, the management of minor ailments is extensive but unstructured and without specific protocols, with great variation in anamnesis, counseling, and recommendations for self-limiting problems (Akpan et al., 2021; Yusuff et al., 2021). Therefore, the management of self-limiting problems in pharmacies, when not guided by clinical guidelines, tends to be variable and often inadequate. Guidelines and protocols improve screening, safety, and standardization of care, but require ongoing training to be effective.

This study has limitations, including its cross-sectional design, reliance on self-reported data subject to recall or social desirability biases, and non-probabilistic online sampling, which introduces potential self-selection bias toward pharmacists interested in prescribing. Although the sample spans all 27 Brazilian federal units and includes non-prescribing professionals (26.1%), findings cannot be directly generalized to all compounding pharmacists in Brazil. Additionally, because recruitment occurred through institutional channels, professional social media platforms, and messaging groups, the precise number of pharmacists reached or who viewed the invitation could not be determined. Consequently, a formal response rate could not be calculated, which is a recognized limitation of web-based surveys using open dissemination strategies. While commercial incentives were identified, we did not assess whether pharmacies implemented formal mechanisms or ethical guidelines to mitigate practice-level conflicts of interest, warranting future research into clinical governance models that ensure financial independence. Finally, the survey did not evaluate whether pharmacists prescribe for health conditions outside their perceived clinical competence or in scenarios with limited scientific evidence, highlighting the need for future studies on prescribers’ decision-making boundaries and clinical governance.

The study presents results that facilitate the understanding of pharmaceutical prescribing in Brazilian compounding pharmacies, highlighting both its potential and limitations. The picture suggests that, despite regulatory support and professional interest, there are significant barriers that need to be addressed to strengthen this clinical practice in a safe, effective, and sustainable manner.

This study characterized the profile of pharmacists working in compounding pharmacies in Brazil to understand the dynamics of pharmaceutical prescribing. It showed that prescribing is a relatively widespread activity among these professionals, especially in the management of self-limiting health problems. However, the findings reveal significant gaps between the practice and its formals documentation, as well as limitations related to specific training, adequate clinical infrastructure, and incentives for its consolidation. Organizational factors still appear to be significant barriers to the expansion of this activity. Despite these challenges, professionals recognize the positive impacts of pharmaceutical prescribing, especially in improving patients’ quality of life, promoting the rational use of medicines, and enhancing the clinical role of pharmacists. This shows that pharmaceutical prescribing in compounding pharmacies has strategic potential to strengthen healthcare in Brazil, provided it is accompanied by advances in clinical training, service structure, legal certainty, and the development of evidence-based clinical guidelines.

## Data Availability

The original contributions presented in the study are included in the article/Supplementary Material, further inquiries can be directed to the corresponding author.

## References

[B1] Akpan R. M. Udoh E. I. Akpan S. E. Ozuluoha C. C. (2021). Community pharmacists’ management of self-limiting infections: a simulation study in Akwa Ibom State, South-South Nigeria. Afr. Health Sci. 21 (2), 576–584. 10.4314/ahs.v21i2.12 34795710 PMC8568250

[B2] Alghamdi K. S. Petzold M. Ewis A. A. Alsugoor M. H. Saaban K. Hussain-Alkhateeb L. (2023). Public perspective toward extended community pharmacy services in sub-national Saudi Arabia: an online cross-sectional study. PLoS One 18 (10), e0280095. 10.1371/journal.pone.0280095 37796778 PMC10553341

[B3] Alrasheed M. A. Alfageh B. H. Almohammed O. A. (2024). Privacy in community pharmacies in Saudi Arabia: a cross-sectional study. Healthcare 12 (17), 1740. 10.3390/healthcare12171740 39273764 PMC11394820

[B4] Brandão M. F. B. Santos F. J. P. (2025). Pharmaceutical prescription in the SUS: expanding access and transforming care. Revista. Científica Eletrônica CRF-BA 4 (1), e04012501. 10.70673/rcecrfba.v4i1.66

[B5] Briskey D. Malfa G. A. Rao A. (2022). Effectiveness of “Moro” blood Orange *Citrus sinensis* osbeck (Rutaceae) standardized extract on weight loss in overweight but otherwise healthy men and women—A randomized double-blind placebo-controlled study. Nutrients 14 (3), 427. 10.3390/nu14030427 35276783 PMC8838101

[B6] Campos C. M. Gallo R. M. Da S. G. H. Lima F. Holanda D. F. (2025). Effect of Moro orange juice extract supplementation in weight management in adults: a systematic review and meta-analysis. Nutr. Health 32, 455–464. 10.1177/02601060251374379 40956687

[B7] Cope L. C. Abuzour A. S. Tully M. P. (2016). Nonmedical prescribing: where are we now? Ther. Adv. Drug Saf. 7 (4), 165–172. 10.1177/2042098616646726 27493720 PMC4959632

[B8] Dagnew E. M. Sendekie A. K. Tsega W. Mekonnen B. A. Getachew M. (2025). Extemporaneous dermatological compounding in hospital pharmacies, Northwest Ethiopia. Front. Public Health 13, 1486936. 10.3389/fpubh.2025.1486936 40520300 PMC12162544

[B9] De Souza M. F. R. de Sena M. P. M. Oliveira C. M. Sales C. A. de Melo R. B. C. de Sena L. W. P. (2022). Analysis of the clinical practice of the pharmacist in a community pharmacy: a cross-sectional study from Brazil. Pharm. Pract. 20 (2), 2658. 10.18549/pharmpract.2022.2.2658 35919800 PMC9296079

[B10] Dhital R. Sakulwach S. Robert G. Vasilikou C. Sin J. (2022). Systematic review on the effects of the physical and social aspects of community pharmacy spaces on service users and staff. Perspect. Public Health 142 (2), 77–93. 10.1177/17579139221080608 35274562 PMC8918882

[B11] Dias K. L. F. Frey J. A. Marquez C. O. (2020). The advantages of compounded medicines vs. manufactured medicines. Rev. Ibero-Am. Humanit. Sci. Educ. 6 (12), 10. 10.29327/217514.6.12-29

[B12] Elshazly M. Jawad S. Ahmed A. ElGeed H. Yusuff K. B. (2025). Enablers and barriers to community pharmacists’ readiness to implement deprescribing of inappropriate medications for older adults in Qatar. PLoS One 20 (1), e0316363. 10.1371/journal.pone.0316363 39883709 PMC11781633

[B13] Federal Council of Pharmacy (2013a). Regulates the Clinical Duties of the Pharmacist and Provides Other Measures: Resolution No. 585. Brasília: CFF. Available online at: https://www.cff.org.br/userfiles/file/resolucoes/585.pdf (Accessed February 2, 2026).

[B14] Federal Council of Pharmacy (2013b). Regulates Pharmaceutical Prescription and Provides Other Measures: Resolution No. 586. Brasília: CFF. Available online at: https://www.cff.org.br/userfiles/file/resolucoes/586.pdf (Accessed February 2, 2026).

[B15] Federal Council of Pharmacy (2025). Official Gazette of the Union: Resolution No. 5. Brasília: CFF. Available online at: https://www.in.gov.br/en/web/dou/-/resolucao-n-5-de-20-de-fevereiro-de-2025-617962146 (Accessed February 2, 2026).

[B16] Federal Council of Pharmacy (2026). Statistics. Brasília: CFF. Available online at: https://site.cff.org.br/estatistica (Accessed February 3, 2026).

[B17] Ferres G. H. M. Lima E. C. A. Silva MJCL Silva M. F. A. Araújo A. M. C. Ferreira F. L. F. (2025). Pharmaceutical care in compounding pharmacies: anintegrative review of Brazilian literature. Braz J. Implantol. Health Sci. 7 (6), 1233–1255. 10.36557/2674-8169.2025v7n6p1233-1255

[B18] Freitas E. M. Silva W. C. Marques D. Skrivan A. G. Coelho A. L. Corrêa D. S. (2022). Pharmaceutical prescription: a new perspective in clinical management. Rev. Científica FAMAP 3(3). Available online at: https://revistacientifica.faculdadefamap.edu.br/revista/article/view/36 (Accessed February 2, 2026).

[B19] Garabeli A. A. Benetoli A. Halila G. C. Machinski I. Tonin F. S. Fernandez-Llimos F. (2022). Mapping community pharmacy services in Brazil: a scoping review. Braz J. Pharm. Sci. 58, e20851. 10.1590/s2175-97902022e20851

[B20] Ghabour M. Morris C. Wilby K. J. Smith A. J. (2023). Pharmacist prescribing training models in the United Kingdom, Australia, and Canada: snapshot survey. Pharm. Educ. 23 (1), 100–108. 10.46542/pe.2023.231.100108

[B21] Hattingh L. Emmerton L. Tin P. Green C. (2016). Utilization of community pharmacy space to enhance privacy: a qualitative study. Health Expect. 19 (1), 144–154. 10.1111/hex.12401 PMC5152744 26332335

[B22] Health Canada (2009). Policyon Manufacturing and Compounding Drug Products in Canada (POL-0051). Ottawa: Health Canada. Available online at: https://www.canada.ca/en/health-canada/services/drugs-health-products/compliance-enforcement/good-manufacturing-practices/guidance-documents/policy-manufacturing-compounding-drug-products.html (Accessed February 2, 2026).

[B23] Hussain R. Babar Z. U. D. (2023). Global landscape of community pharmacy services remuneration: a narrative synthesis of the literature. J. Pharm. Policy Pract. 16 (1), 118. 10.1186/s40545-023-00626-0 37814349 PMC10561514

[B24] Izidoro E. S. S. (2025). Pharmaceutical prescription. Inova Saúde 15 (1), 128–146. 10.18616/inova.v15i1.5287

[B25] Júnior N. N. Rodrigues Ê. F. (2023). Relevant aspects in drug production management in a compounding pharmacy. Adv. Glob. Innov. Technol. 2 (1), 82–90. 10.29327/2384439.2.1-9

[B50] Karia A. M. Chen L. Norman R. Robinson S. Griffiths R. W. Sim T. F. (2024). Barriers and facilitators affecting the implementation of community pharmacist-led professional services in Organisation for Economic Co-operation and Development member countries: a scoping review in the context of expanded scope. Int. J. Pharm. Pract. riaf120. 10.1093/ijpp/riaf120 41359362

[B26] Kim W. Y. Baek A. Kim Y. Suh Y. Lee E. Lee E. (2024). The impact of real-time documentation of In-Hospital medication changes on preventing undocumented discrepancies at discharge and ImprovingPhysician-Pharmacist communication: a retrospective cohort study and survey. J. Multidiscip. Healthc. 17, 2999–3010. 10.2147/JMDH.S460877 38948395 PMC11214548

[B27] López L. C. V. Gallegos D. C. (2022). Pharmacy practice in Latin America: a review of published literature, 2017–2021. DrugsContext 11. 10.7573/dic.2022-3-4 PMC9281972 35912000

[B28] Lugo G. B. Vera Z. C. Aguilar-Rabito A. Samaniego L. R. Larroza G. M. M. (2019). Barriers preventing the effective implementation of pharmaceutical care. ARS Pharm. 60 (4), 199–204. 10.30827/ars.v60i4.9403

[B29] Mastroianni P. C. Forgerini M. Santos A. C. S. Martha A. C. M. Mata B. P. M. Rodrigues E. (2023). Pharmaceutical care and prescription. Marília: Oficina Universitária. 10.36311/2023.978-65-5954-353-3

[B30] Mesbahi Z. Piquer-Martinez C. Benrimoj S. I. Martinez-Martinez F. Amador-Fernandez N. Zarzuelo M. J. (2025). Pharmacists as independent prescribers in community pharmacy: a scoping review. Res. Soc. Adm. Pharm. 21 (3), 142–153. 10.1016/j.sapharm.2024.12.008 39732537

[B31] Milani G. J. Damasceno L. T. Tiguman G. M. B. Aguiar P. M. (2024). Assessment of the implementation of pharmacist prescribing: challenges and pathways for ambulatory practice. Res. Soc. Adm. Pharm. 20 (9), 870–879. 10.1016/j.sapharm.2024.05.002 38762366

[B51] Minghetti P. Pantano D. Gennari C. G. M. Casiraghi A. (2014). Regulatory framework of pharmaceutical compounding and actual developments of legislation in Europe. Health Policy 117, 328–333. 10.1016/j.healthpol.2014.07.010 25110297

[B32] Nardon F. R. Salvi C. P. P. Souza L. B. Pasotti D. V. Júnior A. S. (2024). Profile of herbal medicines dispensed in a pharmacy in the city of Poços de Caldas – MG. Rev. Fitos 18, e1417. 10.32712/2446-4775.2024.1417

[B33] Nassur P. L. Forgerini M. Mastroianni P. C. Lucchetta R. C. (2020). Clinical pharmacy services in Brazil, particularly cardiometabolic diseases: a systematic scoping review and meta-analyses. Pharm Pract. 18 (4), 2131. 10.18549/pharmpract.2020.4.2131 33294063 PMC7699830

[B34] National Association of Compounding Pharmacists (2026). Sectoral Overview. São Paulo: ANFARMAG. Available online at: https://anfarmag.org.br/panorama-setorial/ (Accessed February 3, 2026).

[B35] National Association of Pharmacy Regulatory Authorities (2015). Model Standards for Pharmacy Compounding of Non-Hazardous Sterile Preparations. Ottawa: NAPRA. Available online at: https://www.napra.ca/wp-content/uploads/2024/03/NAPRA-Mdl-Stnds-Pharmacy-Compounding-Non-Hazardous-Sterile-Preparations-EN-Nov-2015-Revised-Nov-2016.pdf (Accessed February 2, 2026).

[B36] National Health Surveillance Agency (Brazil) (2016). Collegiate Board Resolution – RDC No. 98, of August 1, 2016. Establishes Criteria and Procedures for the Classification of Drugs as over-the-Counter. Brasília: ANVISA. Available online at: https://bvsms.saude.gov.br/bvs/saudelegis/anvisa/2016/rdc0098_01_08_2016.pdf (Accessed February 2, 2026).

[B37] Nobbs S. (2020). In Practice: Guidance for Pharmacist‐prescribers. London: General Pharmaceutical Council. Available online at: https://assets.pharmacyregulation.org/files/2025-04/in-practice-guidance-for-pharmacist-prescribers-updated-april-2025.pdf?VersionId=gDMMsBlPEOdD2uTfSyjVzhutXbJ5RCCg (Accessed June 27, 2026).

[B38] Presidency of the Republic (2013). Provides for the Exercise of Medicine Brazil Law No. 12,842. Brasília: Presidency of the Republic. Available online at: https://www.planalto.gov.br/ccivil_03/_ato2011-2014/2013/lei/l12842.htm (Accessed February 2, 2026).

[B39] Presidency of the Republic (2014). Provides for the Exercise and Supervision of Pharmaceutical Activities Brazil Law No. 13,021. Brasília: Presidency of the Republic. Available online at: https://www.planalto.gov.br/ccivil_03/_ato2011-2014/2014/lei/l13021.htm (Accessed February 2, 2026).

[B40] Ramos D. C. Ferreira L. Santos Júnior G. A. D. Ayres L. R. Esposti C. D. D. (2022). Pharmaceutical prescription: a review on perceptions and attitudes of patients, pharmacists, and other stakeholders. Ciênc Saúde Coletiva 27 (9), 3531–3546. 10.1590/1413-81232022279.19972021 36000642

[B41] Rotta I. Lima T. Tonin F. S. (2023). Role of community pharmacy and pharmacists in self-care in Brazil. Explor. Res. Clin. Soc. Pharm. 10, 100274. 10.1016/j.rcsop.2023.100274 37181500 PMC10173763

[B42] Santana R. S. Reis T. M. (2024). Evidence-Based Pharmacy: Pharmaceutical Care Guidelines in Self-Limiting Health Problems (Rio de Janeiro: Editora Atheneu).

[B43] Santi V. (2015). Pharmacist Profile in Brazil: Report (Brasília: Federal Council of Pharmacy). Available online at: https://catalog.nlm.nih.gov/permalink/01NLM_INST/1fua1rm/alma9917162463406676.

[B44] Santos S. T. S. Albuquerque N. L. Guedes J. P. M. (2022). The risks of self-medication with exempted prescription drugs (MIPs) in Brazil. Res. Soc. Dev. 11 (7), e42211730493.

[B45] Selam M. N. Ababu A. Bayisa R. Abdella M. Diriba E. Wale M. (2022). Prescribing pattern of dermatological compounding in Ethiopia: the case of ALERT Hospital. Integr. Pharm. Res. Pract. 11, 1–8. 10.2147/iprp.s346395 35024353 PMC8747791

[B46] Tischner G. Penteado CVDS Colacite J. (2024). Guaranteeing safety and efficiency in compounding pharmacy: a study on quality control practices. Res. Soc. Dev. 13 (12), e173131247795. 10.33448/rsd-v13i12.47795

[B47] Walpola R. L. Issakhany D. Gisev N. Hopkins R. E. (2024). The accessibility of pharmacist prescribing and impacts on medicines access: a systematic review. Res. Soc. Adm. Pharm 20 (5), 475–486. 10.1016/j.sapharm.2024.01.006 38326207

[B48] Woit C. Yuksel N. Charrois T. L. (2020). Competence and confidence with prescribing in pharmacy and medicine: a scoping review. Int. J. Pharm. Pract. 28 (4), 312–325. 10.1111/ijpp.12595 31876027

[B49] Yusuff K. B. Makhlouf A. M. Ibrahim M. I. (2021). Community pharmacists’ management of minor ailments in developing countries: a systematic review of types, recommendations, information gathering and counselling practices. Int. J. Clin. Pract. 75 (10), e14424. 10.1111/ijcp.14424 34081814

