# Supplementary material for: Pharmaceutical profile and prescribing practices in compounding pharmacies in Brazil: a cross-sectional study
Source: Front Pharmacol. 2026 Aug 28;17:1893112. doi: 10.3389/fphar.2026.1893112 (PMC13561903; doi:10.3389/fphar.2026.1893112)
Supplement: Supplementary file 1 [file DataSheet1.pdf]

Supplementary Table 1

Table S1. Bivariate analyses of predictor variables associated with pharmaceutical prescribing practice and formal documentation in Brazilian compounding pharmacies ($N = 186$).

Predictor Variable	Reference / Comparison Category	Target Outcome	Denominator (n/N)	χ^2	*p*-value	Unadjusted OR	95% CI
**Specific Training**	Training/courses in prescribing vs. No training	Prescribing practice	186	22.71	<0.001	5.87	2.75 – 12.53
**Professional Experience**	>5 years of experience vs. ≤5 years	Prescribing practice	186	—	0.019	2.43	1.16 – 5.09
**Physical Infrastructure**	Private consultation room vs. No private room	Formal documentation of prescribing	136	8.86	0.003	3.29	1.51 – 7.16
**Physical Infrastructure**	Private consultation room vs. No private room	Commission receipt / Service fee collection	186	—	<0.001	4.50	1.87 – 10.81

Notes: N = 186 represents the total sample size of responding pharmacists; n = 136 represents the subgroup of pharmacists who reported actively practicing prescribing. χ^2: Pearson's Chi-square statistic. **OR**: Unadjusted Odds Ratio. **95% CI**: 95% Confidence Interval. Statistical significance set at p< 0.05.
